# Serum and serum-derived extracellular vesicle microRNA signatures linked to neurodevelopmental processes in central precocious puberty

**DOI:** 10.3389/fendo.2026.1829887

**Published:** 2026-07-10

**Authors:** Maria Morrou, Vassos Neocleous, Meropi Toumba, Marios Tomazou, Louiza Potamiti, Maria Zanti, Kyriaki Michailidou, Fotios Mpekris, George M. Spyrou, Andreas Protopapas, Elena Sotiriou, Michalis Iasonides, Nicos Skordis, Pavlos Fanis, Leonidas A. Phylactou

**Affiliations:** 1 Department of Molecular Genetics, Function and Therapy, The Cyprus Institute of Neurology and Genetics, Nicosia, Cyprus; 2 Department of Pediatrics, Pediatric Endocrinology Clinic, American Medical Center 2047, Nicosia, Cyprus; 3 School of Medicine, University of Nicosia, Nicosia, Cyprus; 4 Department of Bioinformatics, The Cyprus Institute of Neurology and Genetics, Nicosia, Cyprus; 5 Department of Cancer Genetics, Therapeutics and Ultrastructural Pathology, The Cyprus Institute of Neurology and Genetics, Nicosia, Cyprus; 6 Department of Biostatistics, The Cyprus Institute of Neurology and Genetics, Nicosia, Cyprus; 7 Department of Pediatrics, Makarios III Hospital, Nicosia, Cyprus; 8 Department of Pediatrics, Limassol General Hospital, Limassol, Cyprus; 9 Department of Pediatrics, Iliaktida Pediatric and Adolescent Medical Center, Limassol, Cyprus; 10 Division of Pediatric Endocrinology, Paedi Center for Specialized Pediatrics, Nicosia, Cyprus

**Keywords:** central precocious puberty, extracellular vesicles, microRNA, neurodevelopment, pubertal regulation, puberty onset

## Abstract

**Background:**

Central precocious puberty (CPP) results from premature activation of the hypothalamic–pituitary–gonadal (HPG) axis. While hormonal mechanisms underlying pubertal initiation are well established, the molecular regulatory processes accompanying altered pubertal timing remain incompletely understood. Circulating microRNAs (miRNAs), detectable either freely or within extracellular vesicles (EVs), represent a molecular layer of post-transcriptional regulation associated with pubertal development.

**Methods:**

Serum samples from female patients diagnosed with CPP and age-matched healthy female controls were analyzed by small RNA sequencing to identify differentially expressed miRNAs. Selected miRNAs were validated by quantitative real-time PCR (RT–qPCR) in an expanded cohort using serum RNA and RNA isolated from serum-derived EVs. Functional enrichment analysis was conducted using experimentally validated miRNA target genes.

**Results:**

Small RNA sequencing identified ten miRNAs with significantly altered expression levels in CPP (adjusted p-value < 0.05, |log_2_FC| > 0.5). Pathway enrichment analysis highlighted biological processes related to neurodevelopment, growth regulation and cellular maturation. RT–qPCR validation confirmed reduced serum expression of miR-125a-5p, miR-125b-5p and miR-99b-5p in CPP patients. All selected miRNAs were detectable in serum-derived EVs. Notably, miR-148a-3p exhibited a statistically significant increase specifically within the EV-associated fraction of CPP samples.

**Conclusions:**

This study provides a comprehensive analysis of the circulating miRNA profile in female patients with CPP. The coordinated alteration of freely circulating and EV-associated miRNAs highlight the potential contribution of miRNA-mediated regulation to premature HPG axis activation and provides a framework for further investigation of the molecular mechanisms underlying pubertal disorders.

## 1 Introduction

Puberty is a critical stage of human development, marking the transition from childhood to sexual and reproductive maturity. Reactivation of the hypothalamic–pituitary–gonadal (HPG) axis is characterized by pulsatile gonadotropin-releasing hormone (GnRH) secretion and subsequent stimulation of luteinizing hormone and follicle-stimulating hormone, leading to gonadal maturation and sex steroid production (1). This hormonal cascade drives the development of secondary sexual characteristics, accompanied by accelerated growth and gonadal maturation (2).

Despite extensive research, the precise molecular mechanisms governing the initiation of puberty have not been fully elucidated (3). Pubertal timing is regulated through a complex interplay of genetic and epigenetic influences, as well as nutritional and environmental factors. Physiological onset typically occurs between 8 and 13 years of age in females and 9 and 14 years of age in males (4). Premature activation of the HPG axis due to early GnRH secretion results in central precocious puberty (CPP), predominantly occurring in girls (5, 6). Clinical evaluation of CPP includes assessment of secondary sexual characteristics using Tanner staging, measurement of basal and stimulated gonadotropin levels, bone age estimation, neuroimaging to exclude central nervous system pathology and genetic testing. CPP is considered a clinically significant condition associated with adverse biological, psychosocial and long-term health outcomes, particularly affecting reproductive development, underscoring the need for further investigation into the regulatory mechanisms underlying its pathophysiology (7).

Although CPP is often idiopathic in females, genetic studies have identified mutations in stimulatory and inhibitory regulatory genes such as *KISS1, KISS1R, MKRN3 and DLK1* (8–14). Epigenetic regulation has emerged as another key modulator of physiological pubertal development and its pathological conditions (15, 16). Among the various epigenetic layers, microRNAs (miRNAs) represent a particularly important class of post-transcriptional regulators of pubertal timing (17).

Circulating miRNAs are transported in blood via both vesicular and non-vesicular pathways, suggesting a potential role in systemic signaling and intercellular communication. Specifically, miRNAs can be packaged into extracellular vesicles (EVs) (18). These membrane-bound nanoparticles encapsulate proteins, lipids and nucleic acids, including miRNAs, reflecting features of their cells of origin. Their stability and presence in multiple body fluids have generated interest in EVs as potential non-invasive biomarkers (19).

Recent studies have begun to elucidate the involvement of miRNAs in the regulation of pubertal development (20, 21). In rodent models, miR-200 and miR-155 have been shown to directly modulate GnRH expression or to act via upstream regulatory pathways (22). Another example is miR-30b, which targets Mkrn3 and is upregulated in the hypothalamus during puberty (23). Notably, circulating miR-30b levels also increase during pubertal progression in both boys and girls (24, 25). Furthermore, profiling of hypothalamic miRNAs across juvenile, pubertal and post-pubertal stages has revealed dynamic expression shifts of miRNAs involved in pubertal regulation and epigenetic control (26). However, most evidence derives from animal models, underscoring the need to investigate circulating miRNA expression in human physiological and pathological pubertal states.

In this study, we examined circulating miRNA profiles in female CPP patients and age-matched healthy controls and identified a subset of significantly altered miRNAs associated with neurodevelopmental and growth-related pathways. Furthermore, assessment of miRNA presence in serum-derived EVs revealed distinct patterns of EV-associated miRNA expression, highlighting a previously unexplored compartment of circulating miRNAs in CPP.

## 2 Methods

### 2.1 Participants under study

The study included 22 female CPP patients and 18 female age-matched healthy controls (**Table 1**, **Supplementary Table 1**). All participants were under pediatric endocrinological evaluation with follow-up every three months. Healthy control participants exhibited no clinical signs of secondary sexual development and were confirmed to be prepubertal (Tanner stage 1) at the time of evaluation. CPP was diagnosed clinically according to established criteria, defined as documented breast development and/or pubic or axillary hair development at age younger than 8 years. A gonadotropin-releasing hormone (GnRH/LHRH) stimulation test was performed in 5 of the 22 CPP patients based on clinical indication. In the remaining cases, diagnosis was supported by basal blood tests for FSH, LH and estradiol levels, bone age advancement assessed by x-ray and pelvic ultrasound, in accordance with standard clinical practice. In cases where initial hormonal evaluation was inconclusive, patients were included if subsequent follow-up confirmed progression to CPP. All blood samples were collected prior to initiation of any treatment.

**Table 1:** Clinical and laboratory features of patients.

CPP patient ID	Age at evaluation (years)	Tanner stage*	Basal LH (IU/L)	LH 30 min (IU/L)	LH 60 min (IU/L)	Basal FSH (IU/L)	FSH 30 min (IU/L)	FSH 60 min (IU/L)	Estradiol (pg/ml)	Free T4 (pmol/L)	BA/CA ratio	Pelvic ultrasound^b^	Age at serum sampling (years)	Analysis performed
1	7.83	B3P1A1	0.23	10.61	8.32	1.74	6.97	7.84	25	13.9	1.28	Pubertal	7.83	NGS/RT-qPCR
2	7.42	B2P3A1	0.4	–	–	4	–	–	98	16.3	1.18	Pubertal	8.17	NGS/RT-qPCR
3	7.92	B2P2A1	0.14	–	–	1.24	–	–	47	16.2	0.89	Pubertal	8.25	NGS/RT-qPCR
4	7.58	B4P2A1	7.3	–	–	7.82	–	–	23	–	1.45	Pubertal	7.92	NGS/RT-qPCR
5	7.33	B2P2A1	0.11	–	–	1.29	–	–	6	17.1	1.29	Pubertal	7.33	NGS/RT-qPCR
6	6.58	B2P1A1	0.1	–	–	1.39	–	–	5.4	18.2	1.13	–	7.67	NGS/RT-qPCR
7	8.83	B5P5A2	6.33	–	–	5.3	–	–	25.02	15.2	–	–	8.83	NGS/RT-qPCR
8**	6.83	B2P2A2	0.1	1.6	2.1	1.22	5	7.19	54	17.8	1.00	Pubertal	7.42	NGS/RT-qPCR
9	7.25	B2P1A1	0.6	5.29	7.32	4.2	9.17	13.7	31	15	1.16	Pubertal	7.83	NGS/RT-qPCR
10	5.58	B2P2A1	0.1	–	–	2	–	–	13	14	1.17	Pubertal	6.33	NGS/RT-qPCR
11	8.00	B2P4A3	0.8	–	–	7.6	–	–	39	15	–	–	8.00	NGS/RT-qPCR
12	8.83	B5P3A2	3.69	–	–	6.2	–	–	60	19.9	1.42	Pubertal	8.83	NGS/RT-qPCR
13	7.17	B2P2A2	0.1	1.5	5	1.85	4.8	8.4	–	16.4	1.12	Pubertal	7.42	RT-qPCR
14	8.00	B3P4A3	1	–	–	1	–	–	1.36	–	1.18	Pubertal	8.00	RT-qPCR
15	8.58	B2P2A2	3	–	–	2.8	–	–	6.7	14.9	0.88	Pubertal	9.25	RT-qPCR
16	6.33	B2P2A1	0.1	–	–	1.1	–	–	5	15.3	1.08	–	6.33	RT-qPCR
17	8.58	B2P3A3	–	–	–	3.8	–	–	137.4	12.7	1.18	Pubertal	8.67	RT-qPCR
18	7.67	B3P2A1	0.1	–	–	1.2	–	–	3.6	17.28	1.28	Pubertal	7.67	RT-qPCR
19	8.25	B2P2A2	2.3	–	–	0.9	–	–	10.2	17.9	1.32	Pubertal	8.25	RT-qPCR
20	7.08	B1P3A2	0.1	1	1.39	1.2	3.8	6.35	15	14	1.25	Pubertal	7.17	RT-qPCR
21	7.67	B2P2A2	3.7	–	–	0.15	–	–	<5	–	1.14	–	7.67	RT-qPCR
22	9.50	B3P3A2	1.78	–	–	5.04	–	–	33	14.07	0.95	Pubertal	9.50	RT-qPCR

*Tanner stage. B, breast development; P, pubic hair; A, under arm hair. LH, luteinizing hormone; FSH, follicle-stimulating hormone; T4, thyroxine; TSH, Thyroid-Stimulating Hormone; BA, bone age; CA, chronological age. ^b^Pelvic ultrasound: pubertal determined as with uterus size >3cm and ovarian volume >2.5ml with the presence of multiple follicles. **Sample No. 8 had prepubertal LH/FSH levels at first evaluation and showed pubertal progression at the time of serum sampling.

Exclusion criteria included the presence of peripheral precocious puberty, known endocrine or metabolic disorders, central nervous system abnormalities, chronic systemic diseases, or genetic syndromes affecting growth and pubertal development. Participants receiving hormonal or other treatments known to influence pubertal progression were also excluded. Control participants were additionally excluded if they exhibited any signs of pubertal development or underlying medical conditions.

The study was approved by the Cyprus National Bioethics Committee (Nicosia, Cyprus), and all procedures were conducted in accordance with Law 150(I)/2001 and applicable EU and national data protection regulations, including the General Data Protection Regulation (GDPR) and Law 125(I)/2018. Written informed consent was obtained for all samples associated with identifiable participant data.

### 2.2 Sample collection and genetic analysis

Peripheral blood was collected in serum (BD #369032, BD Biosciences) and EDTA-coated tubes (K2E, BD #367525, BD Biosciences). Genomic DNA was isolated from whole blood using the KingFisher™ Flex Purification System (Thermo Fisher Scientific, Waltham, MA, USA) with the Omega Bio-Tek Mag-Bind Blood & Tissue DNA HDQ 96 kit (Omega Bio-Tek Inc, USA) following the manufacturer’s instructions. DNA concentration and purity were assessed using a NanoDrop ND-1000 spectrophotometer (Thermo Fisher Scientific, Waltham, MA, USA). All samples were negative of any pathogenic variants within the coding region of the *MKRN3* gene.

### 2.3 EV isolation

EVs were isolated from serum using size-exclusion chromatography (SEC). Prior to SEC, serum samples were centrifuged at low speed (1500 x g, 10 min, 4 °C), followed by high speed (10,000 x g, 10 min, 4 °C) to remove cells, cellular debris and large particles. EV isolation was performed by loading 150 μL of serum onto IZON qEV single 70 nm Gen2 columns (Izon Science, Christchurch, New Zealand) and eluted with 0.1 μm-filtered phosphate-buffered saline (PBS). The first four EV-enriched fractions (~680 μL) were pooled and subsequently concentrated to a final volume of 250 μL using 10 kDa centrifugal filters (Amicon Ultra, Millipore, USA) and stored at −80 °C.

### 2.4 EV characterization

#### 2.4.1 Western blotting

EV lysates were prepared by mixing 32 μL EV concentrate with 8 μL of 5× Laemmli buffer and heating at 95 °C for 5 min. Samples were resolved on 10%-15% SDS-PAGE gels and transferred onto activated PVDF membranes (0.45 μm). Membranes were blocked in 3% non-fat dry milk in Tris-buffered saline containing 0.05% Tween-20 (TBST) for 1 hour at room temperature and incubated overnight at 4 °C with primary antibodies diluted in 1% non-fat dry milk in TBST. The following primary antibodies were used: anti-CD63 (1:1000, Abcam #AB134045), anti-Flotillin-1 (1:1000, Abcam #AB133497), anti-Tsg101 (1:1000, Abcam #AB125011), anti-Nucleoporin (1:1000, BD Biosciences #610497) and anti-Cytochrome C (1:1000, BD Biosciences #556432). Membranes were then incubated for 1 h at room temperature with HRP-conjugated secondary antibodies (Goat anti-mouse IgG and goat anti-rabbit IgG, 1:5000, Santa Cruz Biotechnology, USA) diluted in 1% non-fat dry milk in TBST. Signal was detected using enhanced chemiluminescence (Clarity Western ECL, Bio-Rad) and visualized with the Fusion Solo X system (Vilber, Paris, France). Exposure times were optimized for each protein.

#### 2.4.2 Transmission electron microscopy

Samples were deposited onto formvar-coated grids and negatively stained with uranyl acetate. After air drying, the grids were imaged using a transmission electron microscope (Talos L120C, FEI, USA) operated at an accelerating voltage of 120 kV. Micrographs were acquired using a Ceta camera.

#### 2.4.3 Flow cytometry

EVs were analyzed using a two-laser Cytek Northern Lights cytometer equipped with violet (405 nm) and blue (488 nm) lasers, operated with SpectroFlo software (Cytek Biosciences, Fremont, CA, USA). FCMPASS software (version 5.0.10; The Measuring Stick, Eastlands, UK) was used to calibrate the SSC-H parameter into EV diameter (nm), as previously described (27). Instrument calibration employed NIST-traceable polystyrene beads (3000 Series Nanosphere Size Standards; Thermo Scientific, Waltham, MA, USA). Particle diameter and concentration was extrapolated from SSC intensity using Mie scattering theory; lower limit of detection (LOD) corresponded to EV diameter of 73.7 nm. Fluorescent detector settings were optimized using FCMPASS detector optimization tools. Instrument cleanliness was verified prior to acquisition using HPLC-grade water and 0.1 μm-filtered PBS (event rate <1500 events/sec) and performance was confirmed with SpectroFlo QC beads (Cytek Biosciences, Fremont, CA, USA). Data acquisition was performed using an SSC trigger threshold set at 750, a gain of 1500, a low flow rate (15 μL/min) and a recording volume of 15 μL. Data collection commenced after a 20 s stabilization period and the abort rate was maintained below 10%.

For membrane labelling, 2 μL serum was incubated with Vybrant™ CFDA SE Cell Tracer Kit (Invitrogen, Carlsbad, CA, USA) at 50 μM in a total volume of 50 μL for 2 hours at room temperature in the dark. For EV-surface marker detection, 5 μL of serum was incubated with fluorescently conjugated antibodies: PE anti-human CD9 (HI9a; Biolegend, #312106) and anti-human CD81 (5A6; Biolegend, #349512), in a total volume of 50 μL for 30 minutes at room temperature in the dark. Samples were diluted to 150 μL with PBS, subjected to SEC (IZON qEV single 70 nm Gen2 columns; Izon Science, Christchurch, New Zealand) and EV pooled fractions were immediately analyzed. Negative controls were used, including unstained samples, buffer alone, buffer with reagents and detergent-treated stained samples (1% Triton X-100 for 30 minutes), processed under identical conditions in accordance with the MIFlowCyt-EV framework (28).

For quantitative analyses, EV size distribution and particle concentration were determined from calibrated flow cytometry acquisitions. Each sample was analyzed using three independent dilutions and the resulting values were averaged prior to downstream statistical analysis. Particle concentration was presented as particles per μL of serum.

### 2.5 RNA isolation and library preparation

Total RNA, including small RNAs, was isolated from 300 μL of serum using the mirVana PARIS RNA Isolation Kit (Invitrogen, Carlsbad, CA, USA) according to the manufacturer’s instructions. RNA concentration was assessed using a NanoDrop One spectrophotometer (Thermo Fisher Scientific, Waltham, MA, USA). For small RNA sequencing, serum RNA samples from twelve patients and twelve healthy controls were used for miRNA library preparation. Libraries were generated using the QIAseq miRNA Library Kit (Qiagen, Hilden, Germany) following the manufacturer’s protocol. Library quality and fragment size distribution were evaluated using a High Sensitivity D1000 ScreenTape assay on an Agilent TapeStation 2200 (Agilent Technologies, Waldbronn, Germany). Library concentrations were determined using the Qubit dsDNA HS Assay Kit on a Qubit 2.0 Fluorometer (Thermo Fisher Scientific, CA, USA). Individual libraries were normalized to 4 nM and pooled at equimolar concentrations. The pooled library was re-quantified and diluted to a final concentration of 1.6 pM. Sequencing was performed on an Illumina NextSeq 2000 platform using the NextSeq 1000/2000 P2 XLEAP-SBS Reagent Kit (100 cycles).

### 2.6 Bioinformatic analyses

Base calling and demultiplexing was performed onboard the NextSeq sequencer using bcl-convert to obtain reads in.fastq format. The secondary processing including quality control and miRNA mapping was performed using the Nextflow (v24.10.0) (29) nf-core/smrnaseq (v2.4.0) pipeline (30, 31). Specifically, FastQC (v0.12) (32) and fastP (v0.23.4) (33) were used for quality control, adapter trimming and filtering of low base quality scores and length. MirTrace (v.1.0.1) (34) was used for contamination check and removing rRNA, tRNA fragments and other artifacts. UMI deduplication was performed using UMI-tools (35) and UMIcollapse (v1.0.1-1) (36). Next, the reads were mapped against the miRbase database (Release 22.1) (37) and quantified using mirTop (v0.4.28) (38).

Differential expression analysis was performed using the DESeq2 package (39) in R (v4.4) (40) to compare CPP against healthy control samples. Differentially expressed miRNAs (DEMs) were selected by applying an adjusted p-value threshold of <0.05 and a |log2FC| > 0.5. The differential expression results were visualized using ggplot2, EnhancedVolcano and pheatmap packages in R’s BioConductor (41). Next, we used the multiMir package (42) in R, database Version 2.4.0, to fetch all experimentally validated gene targets of the identified DEMs.

Finally, the gene targets were used for functional enrichment. Specifically, clusterprofiler package (43) in R was used to perform Over-Representation Analysis (ORA) against the Gene Ontology (GO) Biological Processes, Molecular Function and Cellular Components database (44) as well as against the KEGG database (45). Enriched terms were selected based on a q-value cutoff of 0.05.

### 2.7 Quantitative real-time PCR

Quantitative reverse transcription PCR (RT-qPCR) was performed to validate the differentially expressed miRNAs identified by NGS analysis. Total RNA was isolated from 300 μL of serum using the mirVana PARIS RNA Isolation Kit (Invitrogen, Carlsbad, CA, USA) and from 200 μL of concentrated EV pooled-fractions, using the Total Exosome RNA and Protein Isolation Kit (Invitrogen, Carlsbad, CA, USA), according to the manufacturer’s instructions. Reverse transcription was carried out using 10 ng of total RNA with the TaqMan MicroRNA Reverse Transcription Kit (Applied Biosystems, Foster City, CA, USA). Real-time PCR was performed in triplicates using TaqMan MicroRNA Assays (Applied Biosystems, Foster City, CA, USA) targeting the following miRNAs: hsa-miR-148a-3p > Assay ID: 000470, hsa-miR-125a-5p > Assay ID: 002198, hsa-miR-125b-5p > Assay ID: 000449, hsa-miR-99b-5p > Assay ID: 000436, hsa-miR-16-5p > Assay ID: 000391, cel-miR-39-3p > Assay ID: 000200. Thermal cycling conditions were as follows: 95 °C for 10 min, followed by 40 cycles of 95 °C for 15 sec and 60 °C for 60 sec. Relative expression levels in serum were normalized to endogenous hsa-miR-16-5p, whereas EV-derived miRNA expression was normalized to the spike-in cel-miR-39-3p. For serum samples, at least two independent RT–qPCR runs were performed per sample and ΔCt values were averaged for statistical analysis. For EV-derived RNA, a single RT–qPCR run was performed per sample due to limited RNA yield.

### 2.8 Statistical analysis

Statistical analyses for RT–qPCR data were performed using ΔCt values. Relative miRNA expression levels were calculated using the comparative Ct method. For each RT–qPCR run, technical replicates were averaged to obtain a single ΔCt value per sample. For serum samples, ΔCt values derived from independent RT–qPCR runs were further averaged prior to downstream statistical analysis. The distribution of ΔCt values was assessed separately within each group using the Shapiro-Wilk test for normality and homogeneity of variances between groups was evaluated using Levene’s test. For each miRNA, comparisons between CPP patients and healthy controls were performed using the unpaired two-tailed two-sample t-test. A p-value < 0.05 was considered statistically significant. To account for multiple testing during RT-qPCR validation, Benjamini-Hochberg false discovery rate (FDR) correction was applied separately to serum and EV-derived miRNA analyses. Adjusted p-value < 0.05 was considered statistically significant. All statistical analyses were performed using the R (v4.4.1) statistical computing language.

## 3 Results

### 3.1 Differential expression profile of serum miRNAs in CPP patients

**Figure 1:**
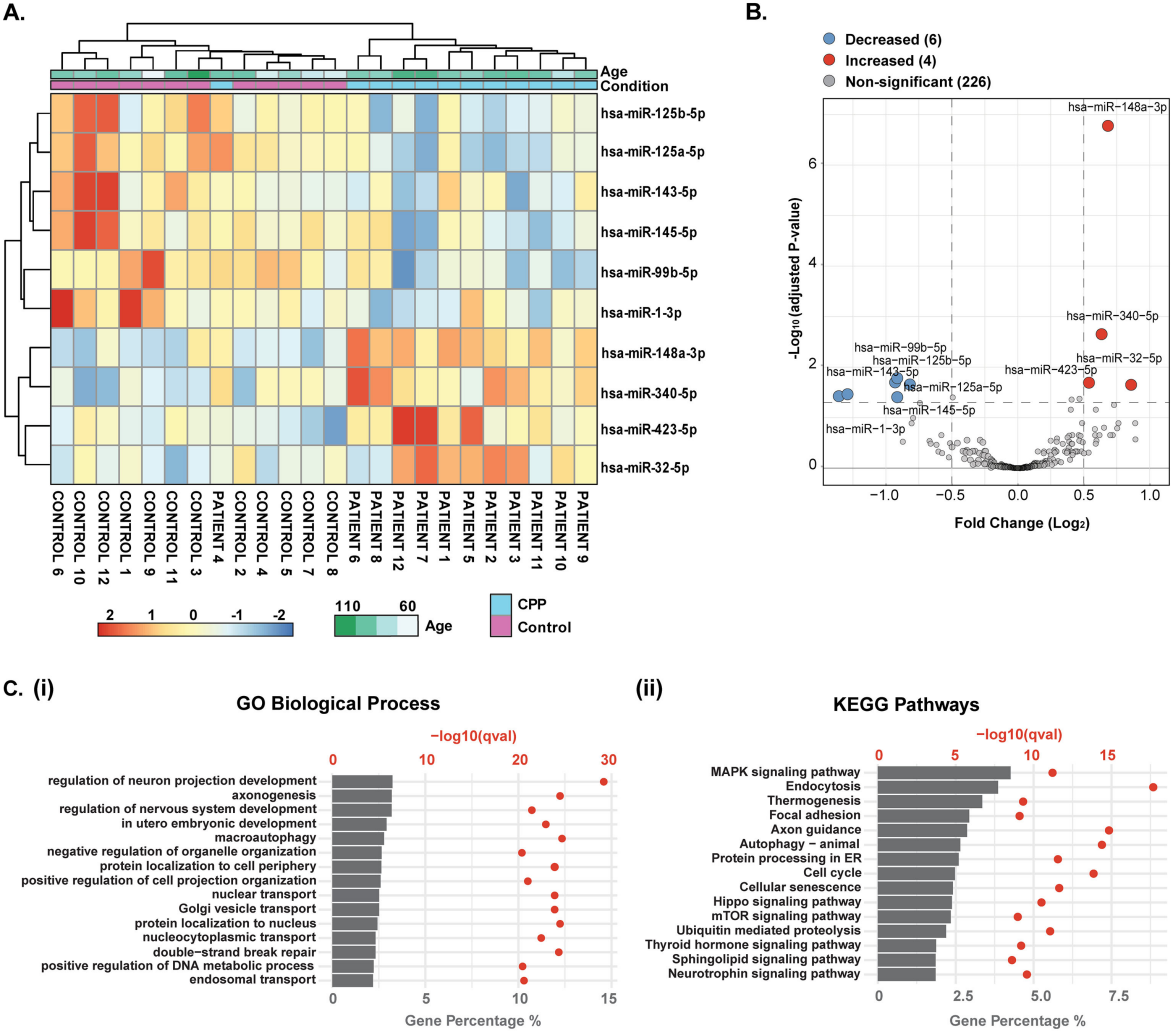
Differential serum miRNA expression and functional enrichment analysis in female CPP patients compared with healthy controls. **(A)** Hierarchical clustering heatmap of the differentially expressed miRNAs. Rows represent miRNAs and columns represent individual samples. Expression values were scaled by row Z-score. Sample annotations indicate age (months) and clinical condition (CPP vs. control). **(B)** Volcano plot of comparison between CPP patients and healthy controls. Dots represent the 236 detected miRNAs. Red dots refer to miRNAs of significantly upregulated levels (adjusted p-value < 0.05, log_2_FC > 0.5) and blue dots to miRNAs of significantly downregulated levels (adjusted p-value < 0.05, log_2_FC < -0.5). Grey dots represent miRNAs of non-significant differential expression. Differential expression analysis was conducted by Bioconductor package DESeq2. **(C)** Functional enrichment analysis of validated target genes of the differentially expressed miRNAs. (i) Gene Ontology (GO) analysis for the biological process. (ii) KEGG pathways analysis. Bars represent the percentage (%) of targeted genes involved in each process or function, relative to the total number of genes targeted by miRNAs. Red dots represent the -log_10_(q-value) of each process or function. FC, Fold Change.

Small RNA sequencing was performed on serum samples from twelve female CPP patients and twelve age-matched healthy controls. Clustering analysis based on serum miRNA expression profiles revealed distinct circulating miRNA expression patterns between groups (**Figure 1A**). Differential expression analysis identified ten miRNAs with significantly altered expression levels in CPP (adjusted p-value < 0.05). Of these, six miRNAs were downregulated in CPP (log_2_FC < −0.5), while four were upregulated (log_2_FC > 0.5) (**Figure 1B**).

To investigate potential associations between differentially expressed miRNAs and clinical characteristics of pubertal development, exploratory correlation analyses were performed within the NGS discovery cohort using the ten differentially expressed miRNAs identified by sequencing (**Supplementary Figure 1**). Clinical parameters included Tanner stage, basal LH, estradiol, free T4 and BA/CA ratio, as indicated in **Table 1**. Overall, correlation coefficients were low and no statistically significant associations were observed between miRNA expression levels and the evaluated clinical parameters.

### 3.2 Pathway enrichment analysis implicates neurodevelopmental pathways associated with pubertal onset

To assess the biological significance of the miRNAs exhibiting altered expression levels in CPP patients, pathway enrichment analysis was performed using experimentally validated target genes. Gene Ontology (GO) Biological Process analysis revealed significant enrichment of terms related to neuronal, reproductive and growth development, including regulation of neuron projection development, axonogenesis, nervous system development and *in utero* embryonic development. Additional enriched biological processes included protein localization, vesicle- and organelle-mediated transport, nuclear transport, as well as DNA metabolic and cell cycle–associated pathways, reflecting broader cellular regulatory mechanisms (**Figures 1C, i**).

KEGG pathway analysis further identified enrichment of signaling pathways involved in cellular growth and differentiation, including MAPK signaling, mTOR signaling, Hippo signaling, neurotrophin signaling, axon guidance and ErbB signaling. Additional enrichment in ubiquitin-mediated proteolysis, endocytosis and protein processing in the endoplasmic reticulum reflects involvement of fundamental cellular regulatory processes (**Figures 1C, ii**).

Notably, several enriched terms were associated with vesicle transport, endosomal trafficking and endocytosis, processes that also contribute to EV biogenesis and cargo sorting. Although these findings do not directly link miRNA alterations to EV regulation, they highlight enrichment of vesicle-related cellular pathways. Overall, the enrichment results indicate that altered circulating miRNAs are associated with neurodevelopmental, growth-related and cellular regulatory pathways relevant to pubertal timing.

### 3.3 Validation of small RNA sequencing results for selected miRNAs

As a next step, miRNAs exhibiting pronounced differential expression levels and relatively high abundance in serum were selected for validation using a second analytical method. Quantitative real-time PCR (RT-qPCR) was performed in an expanded cohort (CPP patients, n = 22; healthy controls, n = 18), including the NGS analysis samples, for the following miRNAs: hsa-miR-125a-5p, hsa-miR-125b-5p, hsa-miR-148a-3p and hsa-miR-99b-5p.

**Figure 2:**
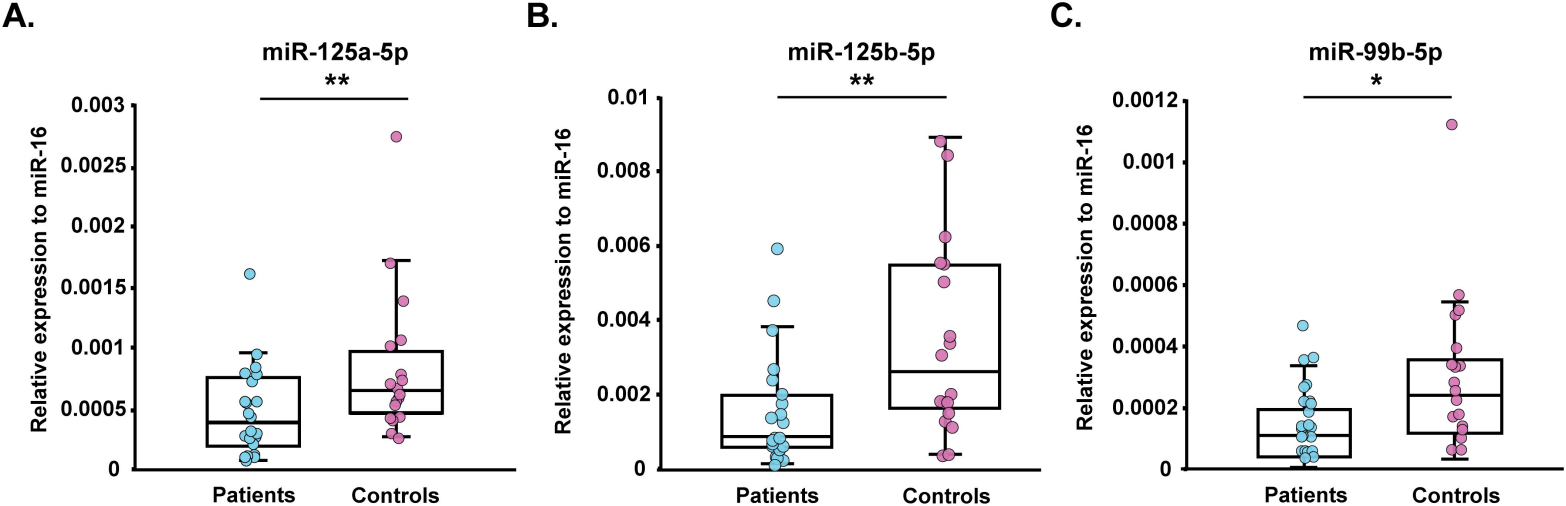
Differential expression of selected miRNAs in female CPP patients and healthy controls measured by quantitative real-time PCR. Serum expression levels of **(A)** miR-125a-5p **(B)** miR-125b-5p and **(C)** miR-99b-5p in CPP patients (n = 22) and healthy controls (n = 18), normalized relative to endogenous miR-16-5p. Box plots display relative expression values (RQ) calculated as 2^ΔCt; boxes represent the interquartile range (IQR) with the median indicated as a horizontal line, whiskers denote 1.5 × IQR and individual data points are overlaid. Statistical comparisons were performed on ΔCt values using an unpaired two-tailed two-sample t-test. Exact p-values: miR-125a-5p, p = 0.0069 (FDR adjusted p = 0.0139); miR-125b-5p, p = 0.0051 (FDR adjusted p = 0.0139); miR-99b-5p, p = 0.0176 (FDR adjusted p = 0.0235). *p < 0.05; **p < 0.01.

RT-qPCR analysis confirmed statistically significant downregulation of miR-125a-5p, miR-125b-5p and miR-99b-5p in CPP patients, consistent with the sequencing results (**Figure 2**). Statistical significance remained following FDR correction for multiple testing. For miR-148a-3p, although the direction of change was concordant with the NGS results, the difference did not reach statistical significance in the validation cohort. Overall, RT-qPCR validation demonstrated concordance with the small RNA sequencing results, supporting the robustness of the observed differential miRNA expression profiles.

### 3.4 Detection of selected miRNAs in serum-derived extracellular vesicles

Given that circulating miRNAs can be transported either freely or within EVs and considering that the pathway enrichment analysis indicated alterations in vesicle transport and endosomal trafficking pathways, we examined whether the serum-identified miRNAs were also present in serum-derived EVs.

RNA was extracted from EVs obtained from control samples (n = 18) and CPP patients (n = 19). The selected miRNAs (miR-125a-5p, miR-125b-5p, miR-99b-5p and miR-148a-3p) were assessed by RT-qPCR and quantified relative to the spike-in control cel-miR-39. All four miRNAs were detectable in EV-derived RNA. For miR-125a-5p and miR-125b-5p, EV-associated expression levels did not differ significantly between CPP patients and healthy controls, whereas for miR-99b-5p a small differential expression between the two groups was observed, displaying a directional trend opposite to that detected in the NGS dataset (**Supplementary Figure 2**).

**Figure 3:**
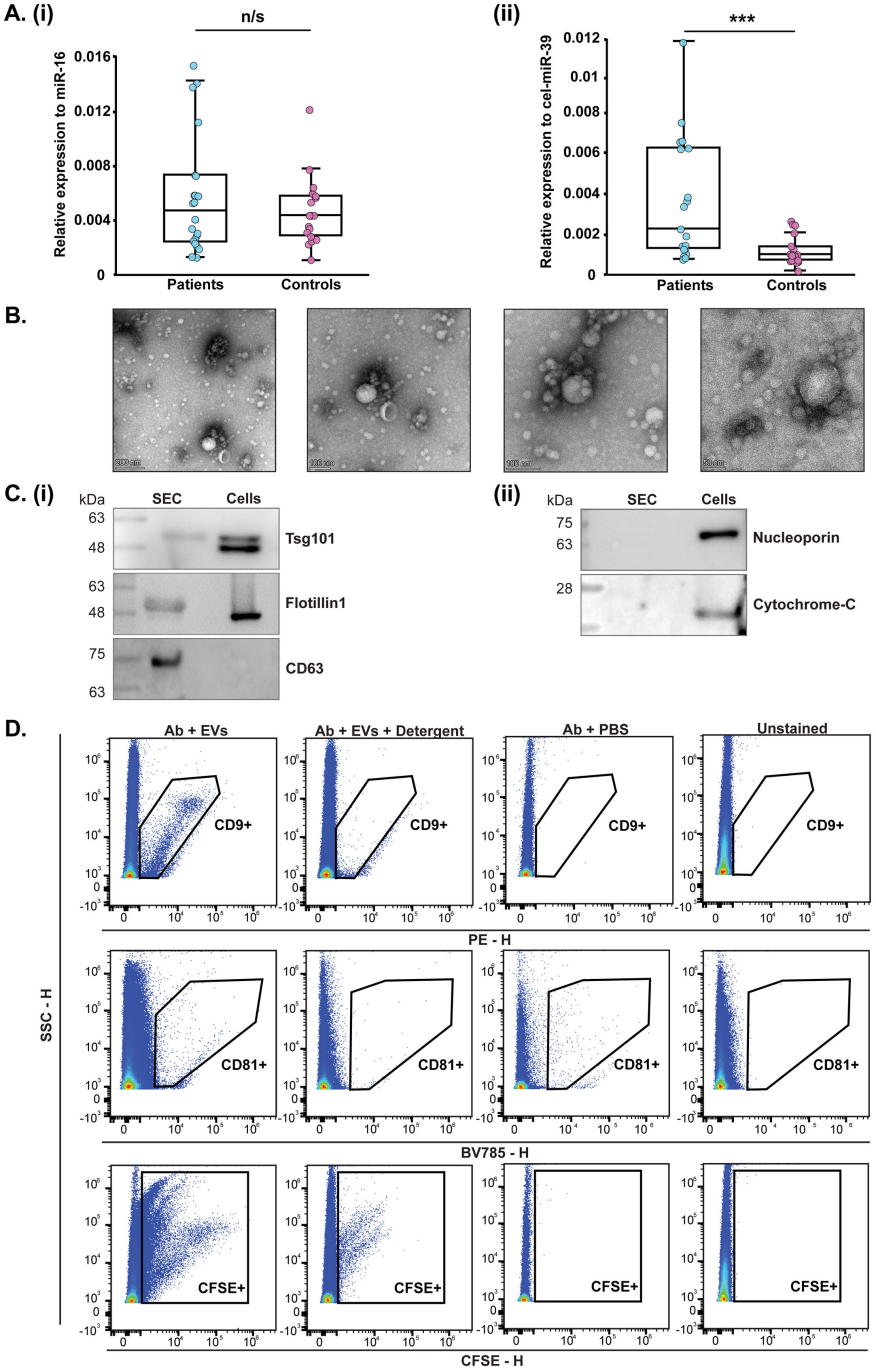
Differential expression of miR-148a-3p in female CPP patients and healthy controls measured by quantitative real-time PCR and characterization of serum-derived extracellular vesicles (EVs). **(A)** Relative expression levels of miR-148a-3p in CPP patients and healthy controls. (i) Serum expression levels normalized relative to endogenous miR-16-5p (CPP, n = 22; control, n = 18). (ii) Serum-derived EV expression levels normalized relative to the spike-in cel-miR-39 (CPP, n = 19; control, n = 18) showing significant upregulation in EVs but not in total serum. Box plots display normalized expression values; boxes represent the interquartile range (IQR) with the median indicated as a horizontal line, whiskers denote 1.5 × IQR and individual data points are overlaid. Statistical comparisons were performed on ΔCt values using an unpaired two-tailed two-sample t-test. Exact p-values: serum, p = 0.7175; EV fraction, p = 0.00076 (FDR adjusted p = 0.0031). ***p < 0.001; ns, non-significant. **(B)** Representative TEM images of negatively stained SEC-isolated EV pools, showing round, membrane-bound vesicles with sizes consistent with small EV populations. **(C)** Western blot analysis of EV‐fraction pools obtained by SEC. Concentrated SEC-derived EV-pools were separated by SDS-PAGE and blotted for (i) EV-associated markers CD63 (~48 kDa), TSG101 (~48 kDa) and Flotillin-1 (~45 kDa) and (ii) cellular contamination markers Nucleoporin (~65 kDa) for nuclear fraction and Cytochrome C (~15 kDa) for cytoplasmic fraction. HEK293T cell lysates were served as positive controls for marker detection. **(D)** Flow cytometry analysis of single EVs. Representative scatter plots of labeled EV‐fraction pools for EV tetraspanins CD9 and CD81 and the membrane-labeling dye CFSE. EV gating was performed relative to detergent-treated EV samples. PBS with dye, unstained EV samples and detergent-treated EV samples were included as negative controls. Detergent treatment (EVs + 0.1% Triton X-100) abolished signal, confirming vesicular origin and staining specificity.

On the other hand, miR-148a-3p, which did not reach statistical significance in serum despite following the same directional trend observed in the NGS dataset, exhibited a statistically significant increase in EV-associated expression levels in CPP patients compared with controls, which remained following FDR correction for multiple testing. EV-associated miR-148a-3p levels were approximately three-fold higher in CPP patients, representing an even greater increase than that observed in whole-serum sequencing data (**Figure 3A**). This observation indicates that the differential expression signal for miR-148a-3p in serum is strongly represented within the EV-associated fraction, whereas for the other miRNAs, the EV-associated compartment did not account for the differential expression observed in whole serum.

To confirm the identity and purity of the isolated vesicles, EVs were characterized using complementary approaches. TEM confirmed the presence of membrane-bound nanoparticles with morphology consistent with EVs (**Figure 3B**). Western blotting verified the enrichment of established EV markers and the absence of cellular contaminants (**Figure 3C**) and the corresponding full-length uncropped blots are provided in **Supplementary Figure 3**. Flow cytometry further demonstrated the presence of EV-associated surface markers (**Figure 3D**). Control analyses showed minimal background in buffer and unstained samples, while detergent treatment of stained EVs resulted in loss of signal, supporting that the detected events represented membrane-enclosed vesicles (**Figure 3D**). Unstained EV samples from CPP patients and healthy controls were also analyzed by calibrated flow cytometry to generate size-distribution profiles and quantitative particle concentration measurements. The mean EV diameter was 106 nm for control samples (n = 18) and 101 nm for CPP patient samples (n = 19). EV size distributions were highly similar between CPP patients and healthy controls, suggesting comparable EV populations in terms of vesicle size (**Supplementary Figure 4A–C**). Quantitative flow cytometric analysis demonstrated a trend toward higher EV particle concentrations in CPP samples compared with controls, however the difference did not reach statistical significance (Mann-Whitney test, p = 0.0656) (**Supplementary Figures 4D**).

## 4 Discussion

Pubertal onset is driven by activation of the hypothalamic–pituitary–gonadal (HPG) axis and coordinated endocrine signaling that regulates peripheral development and promotes reproductive maturation (1). Increasing evidence suggests that circulating regulatory molecules, including microRNAs, contribute to the fine-tuning of pubertal development and neuroendocrine signaling (46, 47). In this context, circulating miRNAs, stable in serum either freely or within EVs, represent potential regulatory components of pubertal timing (48).

In this study, small RNA sequencing identified differential expression of ten circulating miRNAs in females with CPP, indicating coordinated alterations in the circulating miRNA landscape (**Figure 1**). Enrichment analysis of experimentally validated miRNA targets highlighted pathways related to neurodevelopment, growth-related and cellular maturation processes, indicating integration within regulatory networks relevant to pubertal timing. Validation in an expanded cohort confirmed reduced serum levels of miR-125a-5p, miR-125b-5p and miR-99b-5p. In parallel, analysis of serum-derived EVs demonstrated the presence of these miRNAs as EV cargo, with miR-148a-3p exhibiting a pronounced and statistically significant increase in the EV-associated fraction of CPP patients.

Within the broader set of altered miRNAs identified in this study, multiple candidates are linked to regulatory networks of interest. Consistent with this, separate enrichment analyses for each of the validated miRNAs revealed association with growth, neurodevelopment and cellular regulatory pathways (**Supplementary Figure 5**). Members of the miR-125 family, which were downregulated in CPP patients, are associated with pathways involved in cell cycle regulation, intracellular signaling and cellular stress responses, suggesting a potential role in modulating developmental progression. Several studies have demonstrated the role for miR-125a and miR-125b in human neuronal differentiation (49, 50). Similarly, miR-99b, consistently downregulated in CPP, has been implicated in RNA metabolism, chromosome organization and mitotic regulation. Together, these findings reflect broader shifts in transcriptional and post-transcriptional regulatory mechanisms accompanying accelerated maturation. In this context, the validated miRNAs serve as representative components of a wider miRNA network whose collective dysregulation may contribute to the complex growth and developmental phenotype characteristic of CPP.

Beyond overall changes in circulating miRNA abundance, the present findings point to EVs as an additional layer of miRNA compartmentalization in CPP. Although validated miRNAs were detectable in EV fractions, miR-148a-3p exhibited significant enrichment in CPP patients. While the tissue origin and functional consequences of EV-associated miR-148a-3p cannot be determined from the present data, its compartment-specific increase indicates preferential detection within the EV-associated fraction. Further studies will be required to determine whether this reflects selective miRNA packaging, altered EV abundance, or other mechanisms. Notably, GO enrichment analysis identified “*in utero* embryonic development” as the most significantly enriched biological process among predicted miR-148a-3p target genes. This annotation reflects broader developmental programs and highlights the involvement of regulatory networks that may also contribute to maturation and activation of the reproductive axis. In addition, miR-148a-3p has validated targets including *MKRN3 and DLK1*, key inhibitory regulators of pubertal onset and regulation of the HPG axis (51, 52). This association is consistent with the possibility that EV-associated miRNAs reflect molecular pathways relevant to neuroendocrine regulation and premature HPG axis activation, although functional studies will be required to establish the mechanistic relationship.

Collectively, these findings indicate that CPP is accompanied by coordinated alterations in circulating miRNA regulatory networks. The presence of distinct freely circulating and EV-associated signatures is consistent with puberty representing a multisystem developmental transition integrating neuroendocrine activation with peripheral maturation. Rather than serving solely as diagnostic biomarkers, circulating miRNAs may reflect systemic regulatory states linked to developmental, neuroendocrine and reproductive processes associated with altered pubertal timing.

These observations are consistent with previous studies demonstrating that circulating miRNAs are involved in the regulation of pubertal timing and neuroendocrine function. Emerging evidence indicates that miRNA-dependent regulatory mechanisms are essential for the activation of hypothalamic GnRH neurons, which drive the onset of puberty. In particular, specific miRNA networks, including the miR-200 and miR-30 families, have been shown to regulate GnRH transcription and upstream inhibitory pathways such as MKRN3, a key suppressor of pubertal initiation (22, 23, 53).

Within this framework, the miRNAs identified in the present study, several of which are associated with neuronal differentiation, synaptic plasticity and cellular maturation, may reflect regulatory processes relevant to the development and functional activation of hypothalamic networks controlling puberty. Although these specific miRNAs have not been directly implicated in pubertal timing, their association with neurodevelopmental pathways is consistent with the hypothesis that circulating miRNA alterations in CPP may represent downstream signatures of neuroendocrine maturation.

In the present study, EV characterization included quantitative assessment of both EV size distribution and particle concentration using calibrated flow cytometry. Although CPP samples exhibited a trend toward higher EV concentrations, this difference did not reach statistical significance. Nevertheless, EV-associated miRNA expression was normalized using a spike-in control rather than EV particle number. Therefore, the observed differences in EV-associated miRNA levels may reflect differences in EV abundance, selective miRNA enrichment, alterations in EV cargo loading, or a combination of these factors. Future studies incorporating particle-normalized miRNA quantification and additional quantitative EV characterization approaches will help further clarify the relationship between EV abundance and EV-associated miRNA dynamics in CPP.

As an observational study, our analyses identify molecular alterations associated with premature pubertal activation. Circulating miRNA profiles may be influenced by pubertal stage and associated hormonal changes. Two CPP patients had reached menarche at the time of sampling; however, this is unlikely to substantially influence the overall findings of the study. Nevertheless, subtle differences in pubertal progression may contribute to variability in miRNA expression. Consistent with this, exploratory correlation analyses performed within the discovery cohort did not identify significant associations between miRNA expression levels and the evaluated clinical characteristics, although the limited sample size restricted the statistical power of these analyses. An additional consideration relates to the diagnostic heterogeneity inherent to the clinical evaluation of CPP. Although all patients fulfilled established diagnostic criteria, GnRH stimulation testing was performed only when clinically indicated, while diagnosis in the remaining cases relied on a combination of clinical presentation, hormonal measurements, imaging findings and longitudinal follow-up. This approach reflects routine clinical practice but may introduce some variability in disease classification and consequently in the molecular profiles observed.

Another important consideration of the present study is the size of the NGS discovery cohort. Although the principal findings were subsequently evaluated in an expanded RT-qPCR validation cohort, the sequencing analysis was performed in twelve CPP patients and twelve healthy controls, reflecting the challenges associated with recruiting well-characterized pediatric CPP cohorts. While the cohort size may have limited the ability to investigate biological heterogeneity and perform detailed stratification according to pubertal stage, hormonal profile or other clinical characteristics, the independent validation of the principal serum findings in the expanded cohort supports the robustness of the identified miRNA alterations. Larger studies incorporating broader clinical stratification and longitudinal follow-up across defined pubertal stages will be important for further defining the relationship between circulating miRNAs and clinical characteristics of pubertal development.

Overall, the present work defines a distinct circulating and EV-associated miRNA landscape linked to CPP and supports circulating miRNA alterations as a complementary molecular layer accompanying classical endocrine regulation.

## Data Availability

The datasets presented in this study can be found in online repositories. The names of the repository/repositories and accession number(s) can be found in the article/**Supplementary Material**.
