# Supplementary material for: Serum and serum-derived extracellular vesicle microRNA signatures linked to neurodevelopmental processes in central precocious puberty
Source: Front Endocrinol (Lausanne). 2026 Jul 10;17:1829887. doi: 10.3389/fendo.2026.1829887 (PMC13395601; doi:10.3389/fendo.2026.1829887)
Supplement: Supplementary file 1 [file DataSheet1.pdf]

Supplementary Material

Raw sequencing data have been deposited in the European Nucleotide Archive (ENA; accession number PRJEB107731.

Supplementary Table S1. Age of healthy control participants.

Healthy Control	Age at serum sampling (years)
**1**	7.25
**2**	8.00
**3**	9.83
**4**	5.67
**5**	7.17
**6**	7.83
**7**	5.92
**8**	5.58
**9**	5.00
**10**	7.42
**11**	8.17
**12**	8.08
**13**	7.17
**14**	6.75
**15**	7.92
**16**	6.50
**17**	8.67
**18**	6.58

A. Breast Development

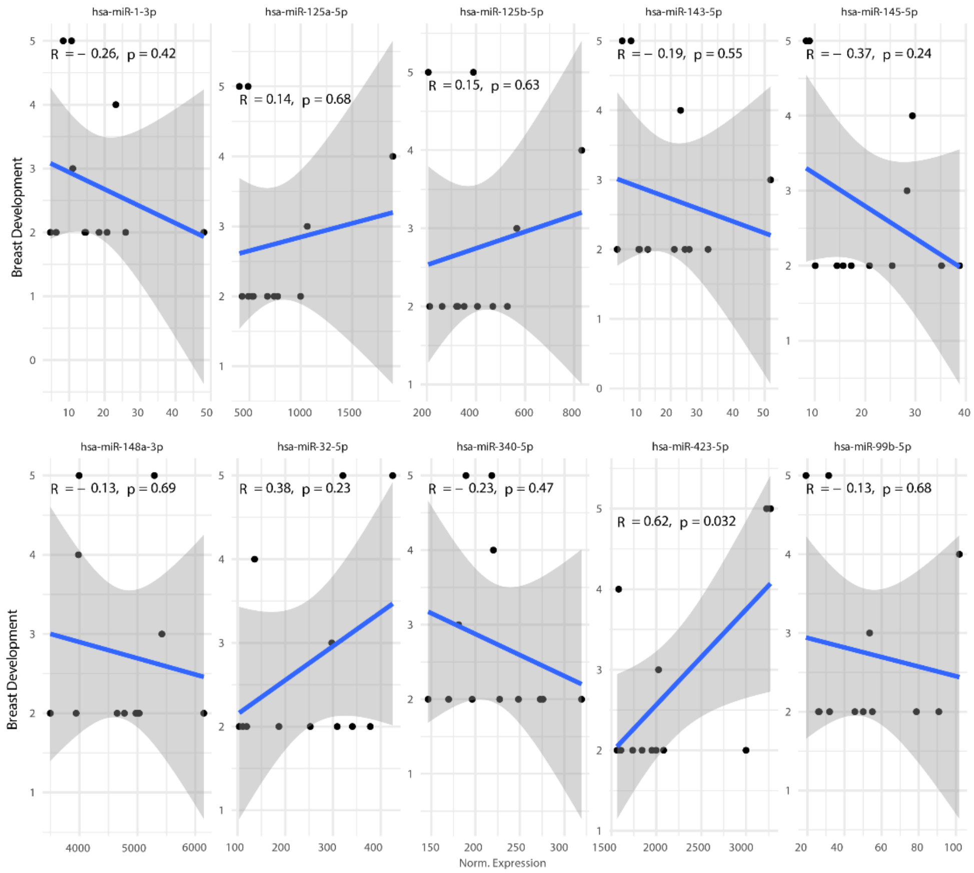

B. Pubic Hair

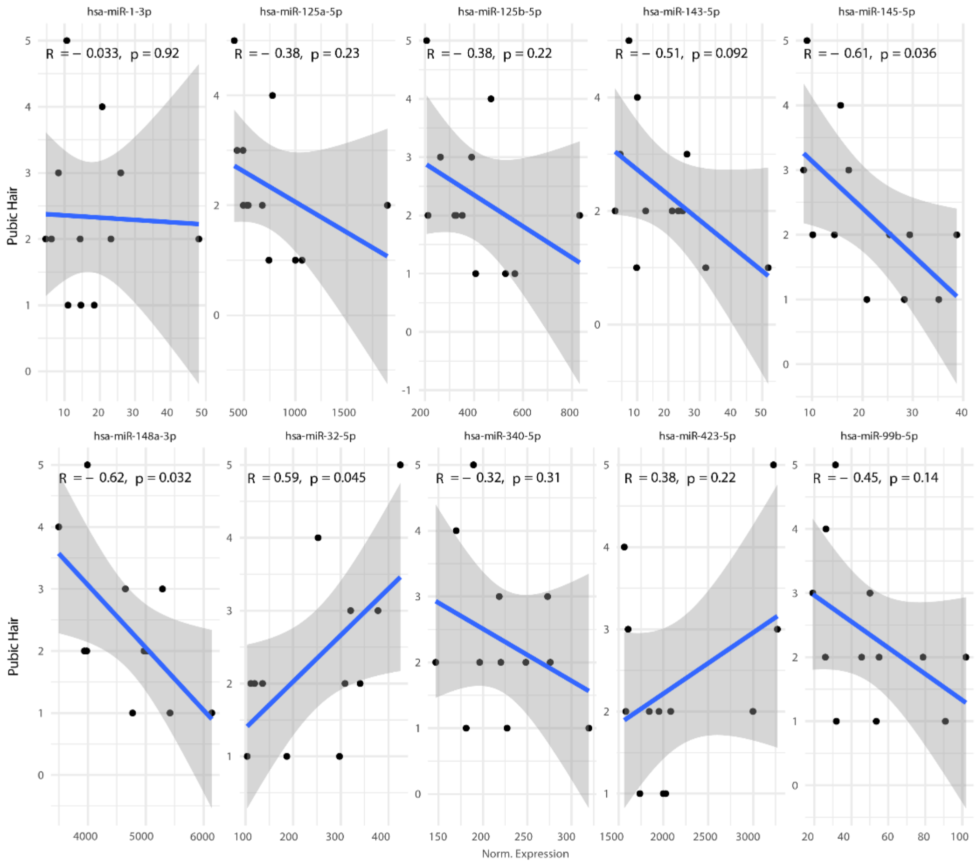

C. Axillary Hair

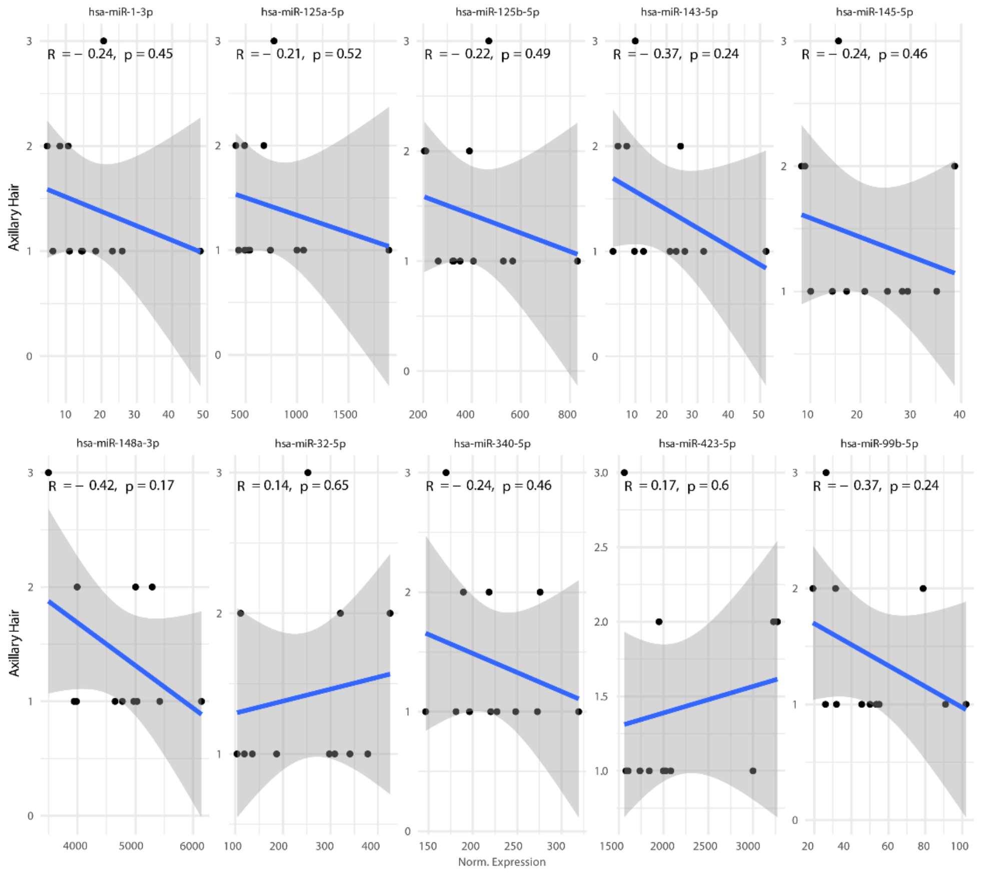

D. Free T4

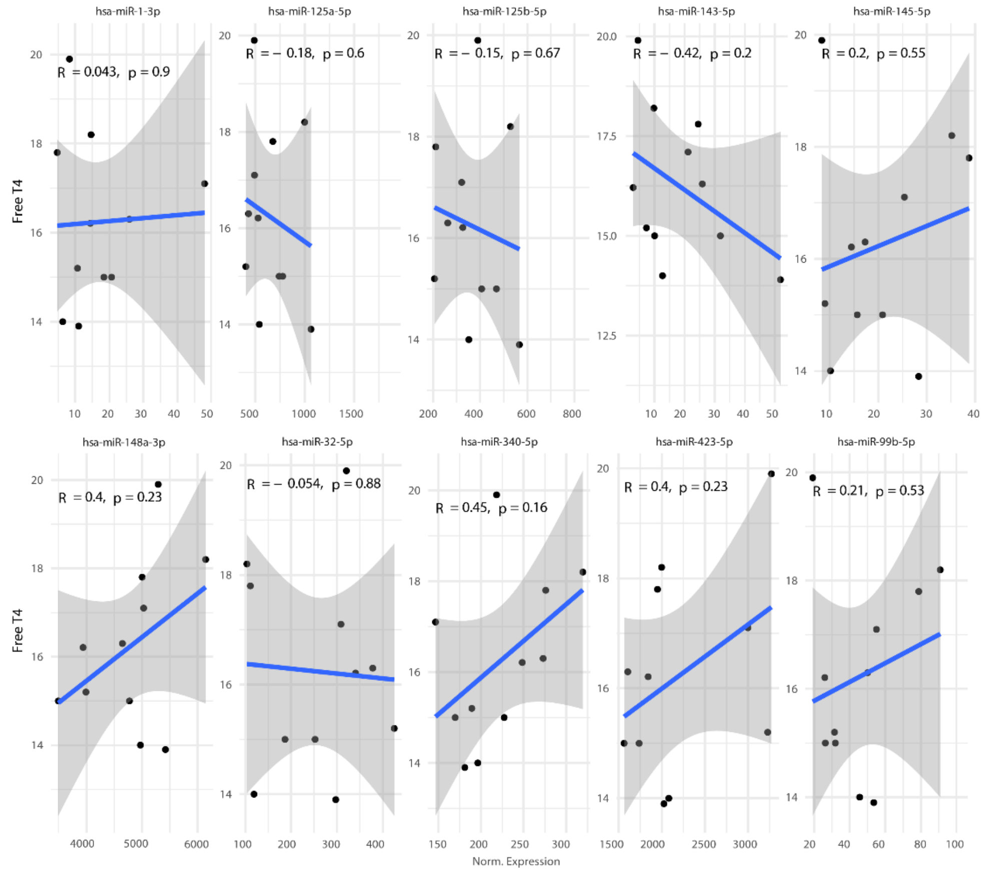

E. Basal LH

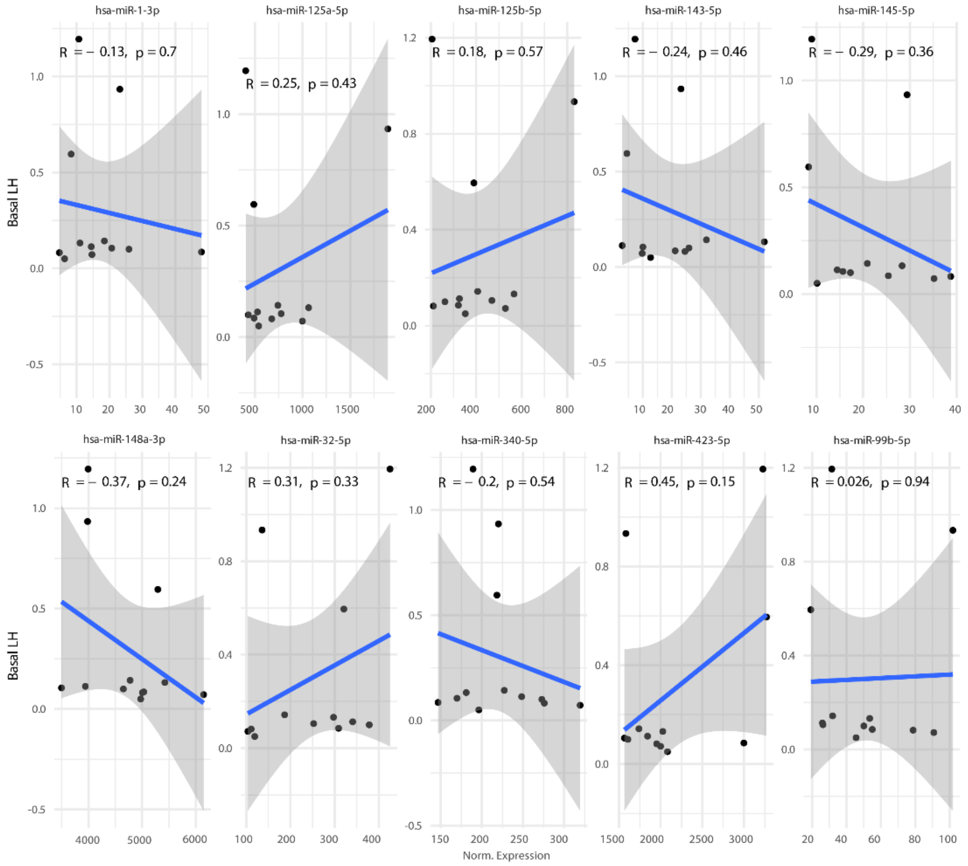

F. Estradiol

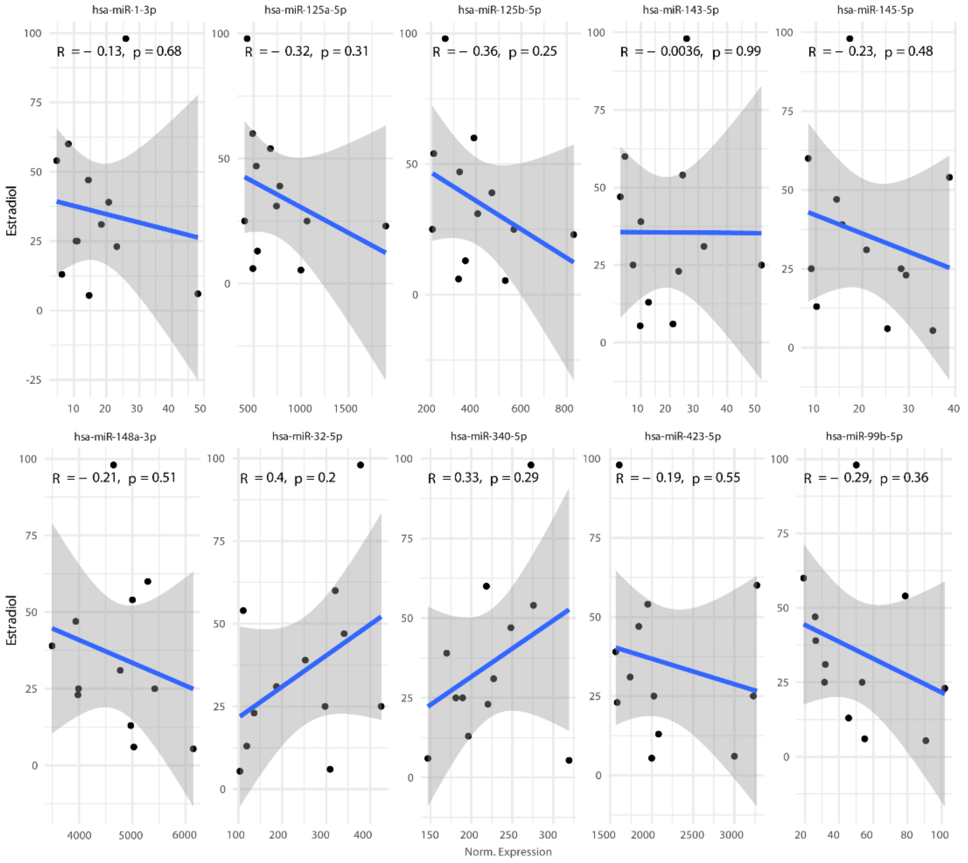

G.

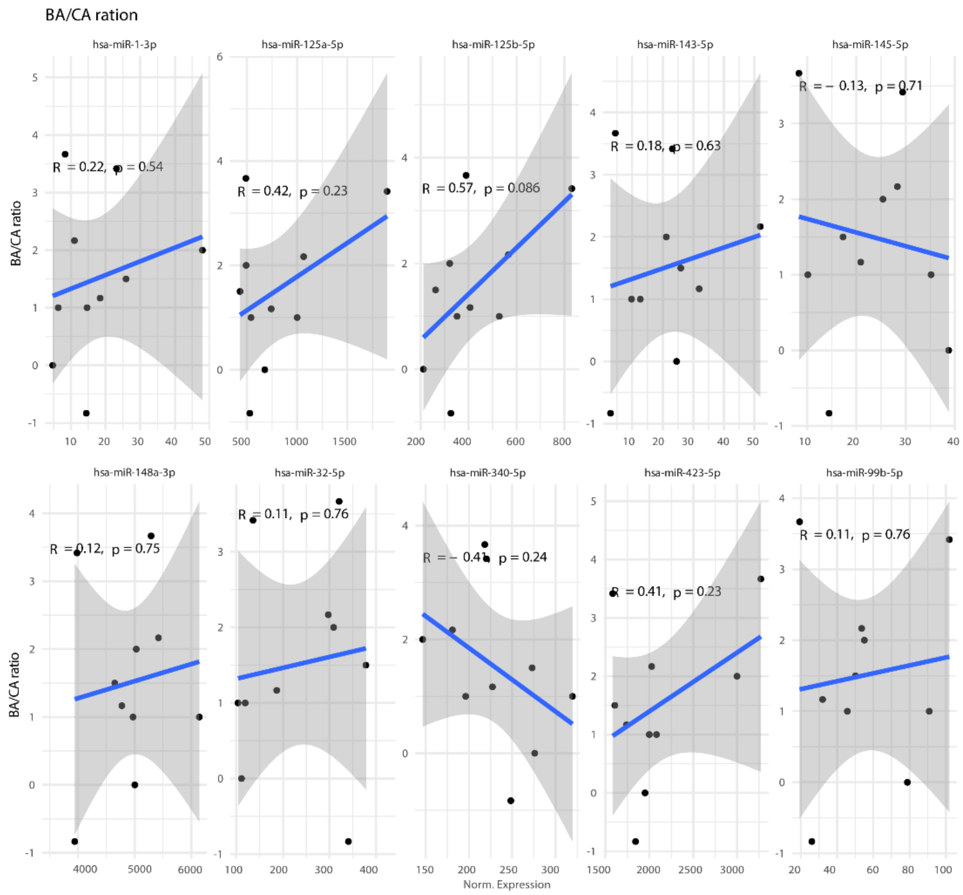

Supplementary Figure S5. Exploratory correlation analysis between differentially expressed miRNAs and clinical characteristics within the CPP cohort.

Spearman correlation analyses were performed between the normalized expression levels of the ten differentially expressed miRNAs identified by small RNA sequencing and clinical parameters available for CPP patients included in the discovery cohort (n = 12). Correlations are shown for **(A)** breast development stage **(B)** pubic hair stage **(C)** axillary hair stage **(D)** free thyroxine (Free T4) (pmol/L) **(E)** basal luteinizing hormone (Basal LH) (IU/L), **(F)** estradiol concentration (pg/ml) and **(G)** bone age-to-chronological age ratio (BA/CA). Each point represents an individual patient. Blue lines indicate fitted regression trends and shaded areas represent the 95% confidence interval. Correlation coefficients (R) and corresponding p-values are shown within each panel.

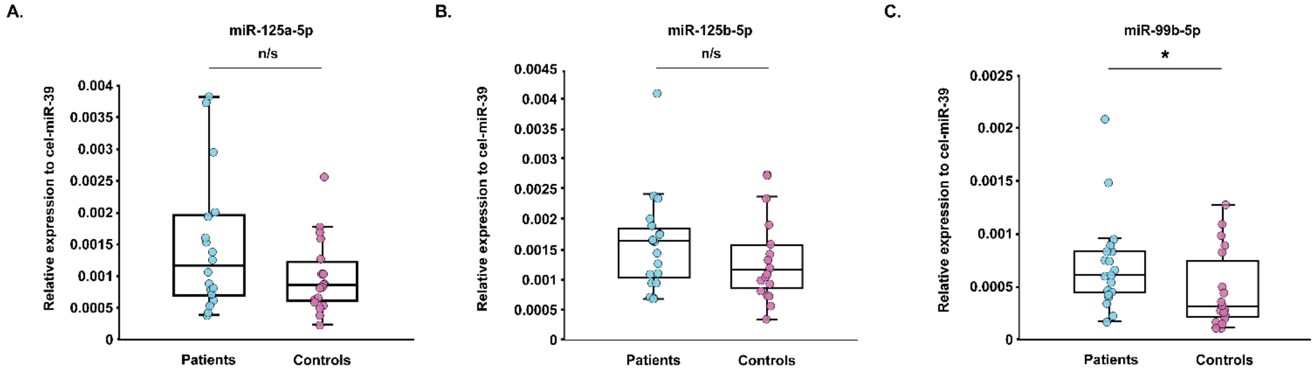

Supplementary Figure S2. Expression of selected miRNAs in serum-derived extracellular vesicles from CPP patients and healthy controls.

Relative expression levels of **(A)** miR-125a-5p (CPP, n = 18; control, n = 18), **(B)** miR-125b-5p (CPP, n = 19; control, n = 18), and **(C)** miR-99b-5p (CPP, n = 19; control, n = 18) in EV-derived RNA, normalized relative to the spike-in control cel-miR-39. Box plots display relative expression values (RQ) calculated as $2^\Delta Ct$; boxes represent the interquartile range (IQR) with the median indicated as a horizontal line, whiskers denote 1.5 × IQR, and individual data points are overlaid. Statistical significance between groups was assessed using an unpaired two-tailed two-sample t-test on ΔCt values (* $p < 0.05$; n/s, non-significant).

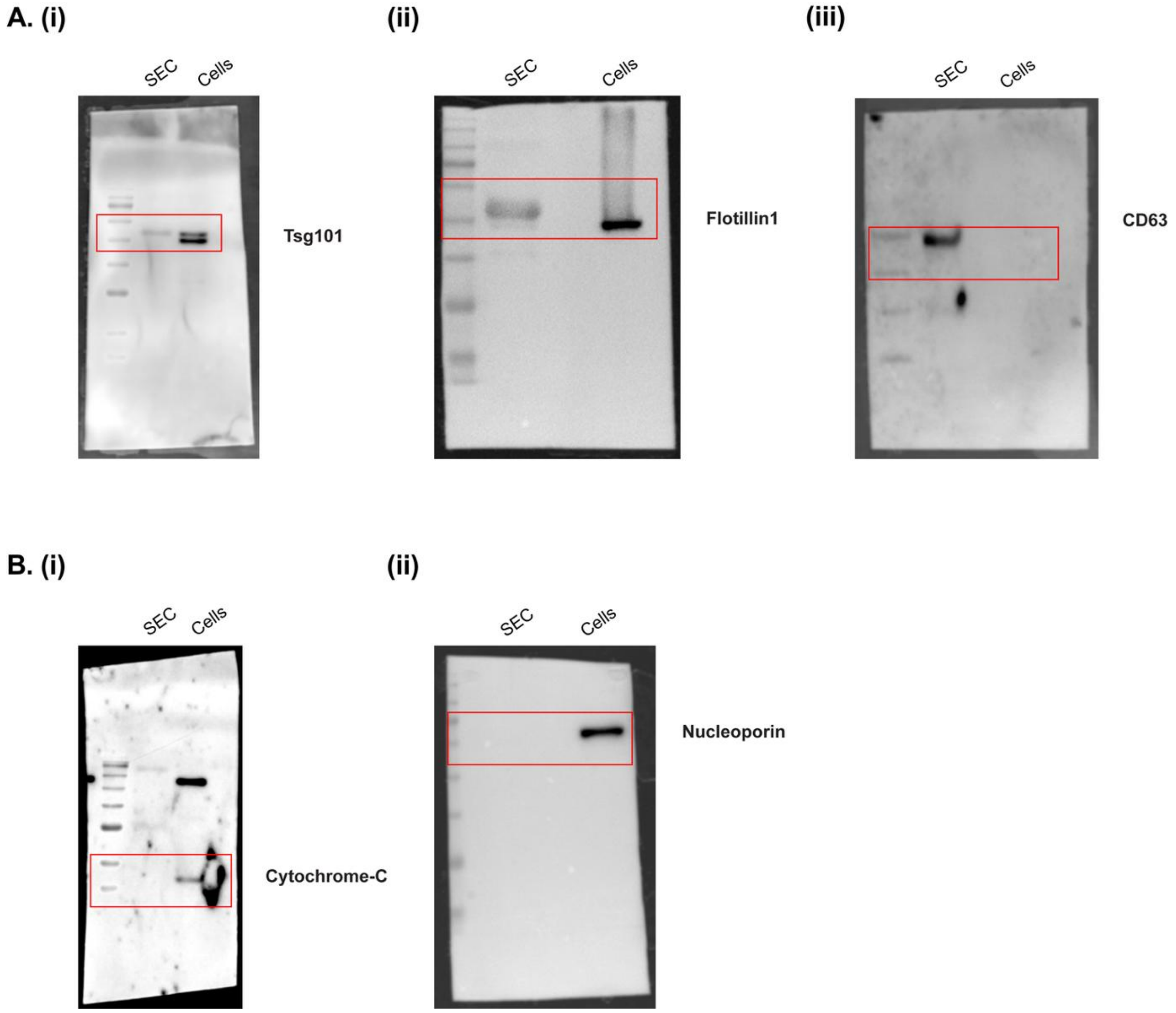

Supplementary Figure S3. Full-size immunoblots used for the characterization of EV-fraction pools obtained by SEC, presented in Figure 3C.

Red rectangles indicate the specific parts of the immunoblots used for generation of Figure 3. **(A)** Detection of EV-associated markers (i) TSG101 (~48 kDa), (ii) Flotillin-1 (~45 kDa) and (iii) CD63 (~48 kDa). **(B)** Detection of cellular contamination markers (i) Cytochrome C (~15 kDa) for cytoplasmic fraction and (ii) Nucleoporin (~65 kDa) for nuclear fraction. HEK293T cell lysates served as positive controls for marker detection.

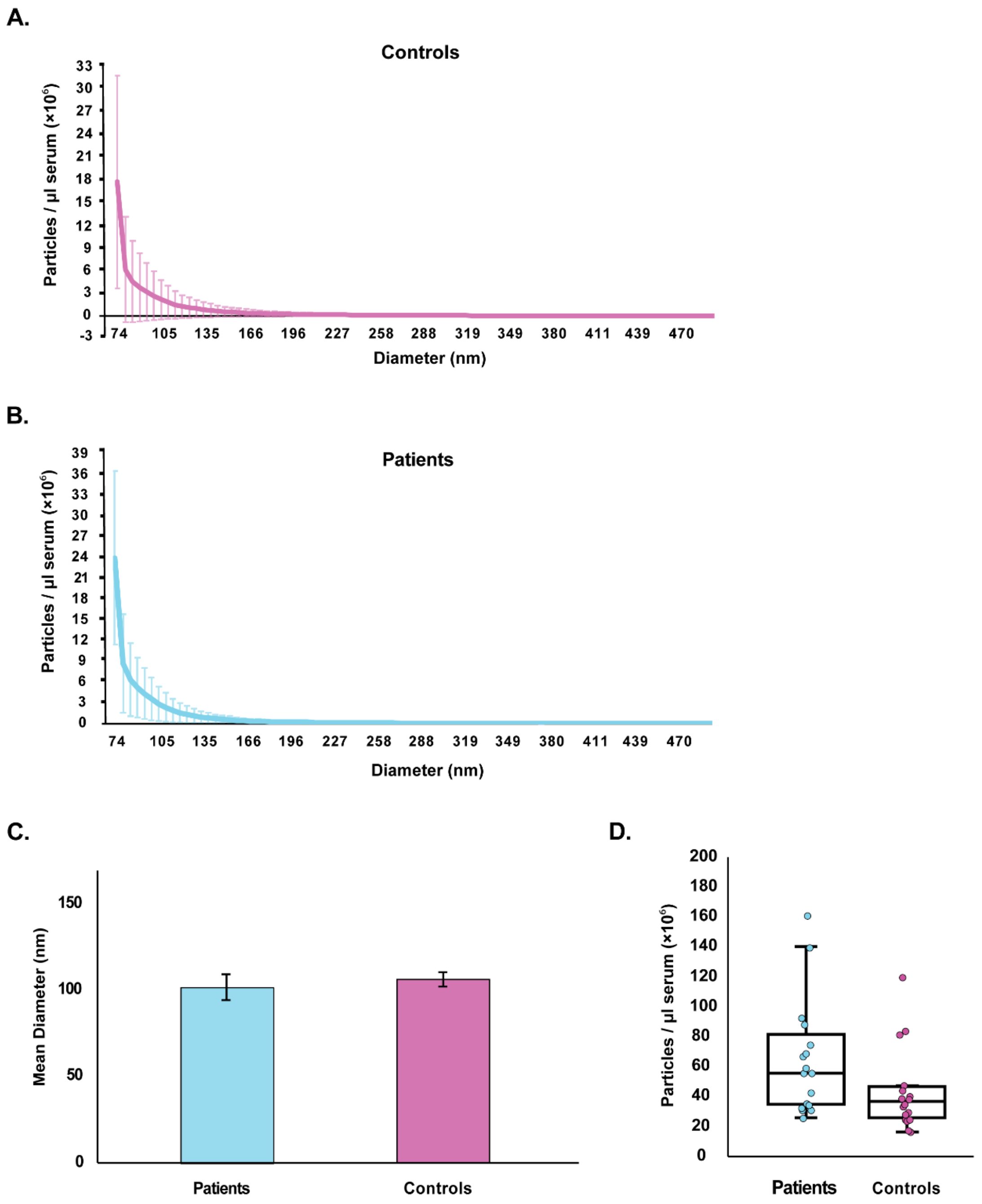

Supplementary Figure 4. Size distribution, mean diameter and particle concentration of serum-derived extracellular vesicles in CPP patients and healthy controls.

(A) Diameter distribution of unstained serum-derived EVs from healthy control samples, showing particle counts per µL of serum across calibrated diameter bins. **(B)** Diameter distribution of unstained serum-derived EVs from CPP patient samples, showing particle counts per µL of serum across calibrated diameter bins. For each sample, three independent dilutions were measured by flow cytometry and averaged prior to group analysis. Error bars in panels A and B represent the standard deviation (SD) across averaged sample values within each group. **(C)** Mean EV diameter in healthy controls (n = 18) and CPP patients (n = 19), calculated from averaged sample values. Bars represent

mean ± SD. **(D)** Particle concentration measured by calibrated flow cytometry and presented as particles per µL of serum. For each sample, three independent dilutions were acquired and averaged prior to statistical analysis. Each point represents the average particle concentration of an individual sample. Box plots display the median and interquartile range. CPP samples exhibited a trend toward higher EV concentrations compared with healthy controls; however, the difference did not reach statistical significance (Mann – Whitney test, p = 0.0656).

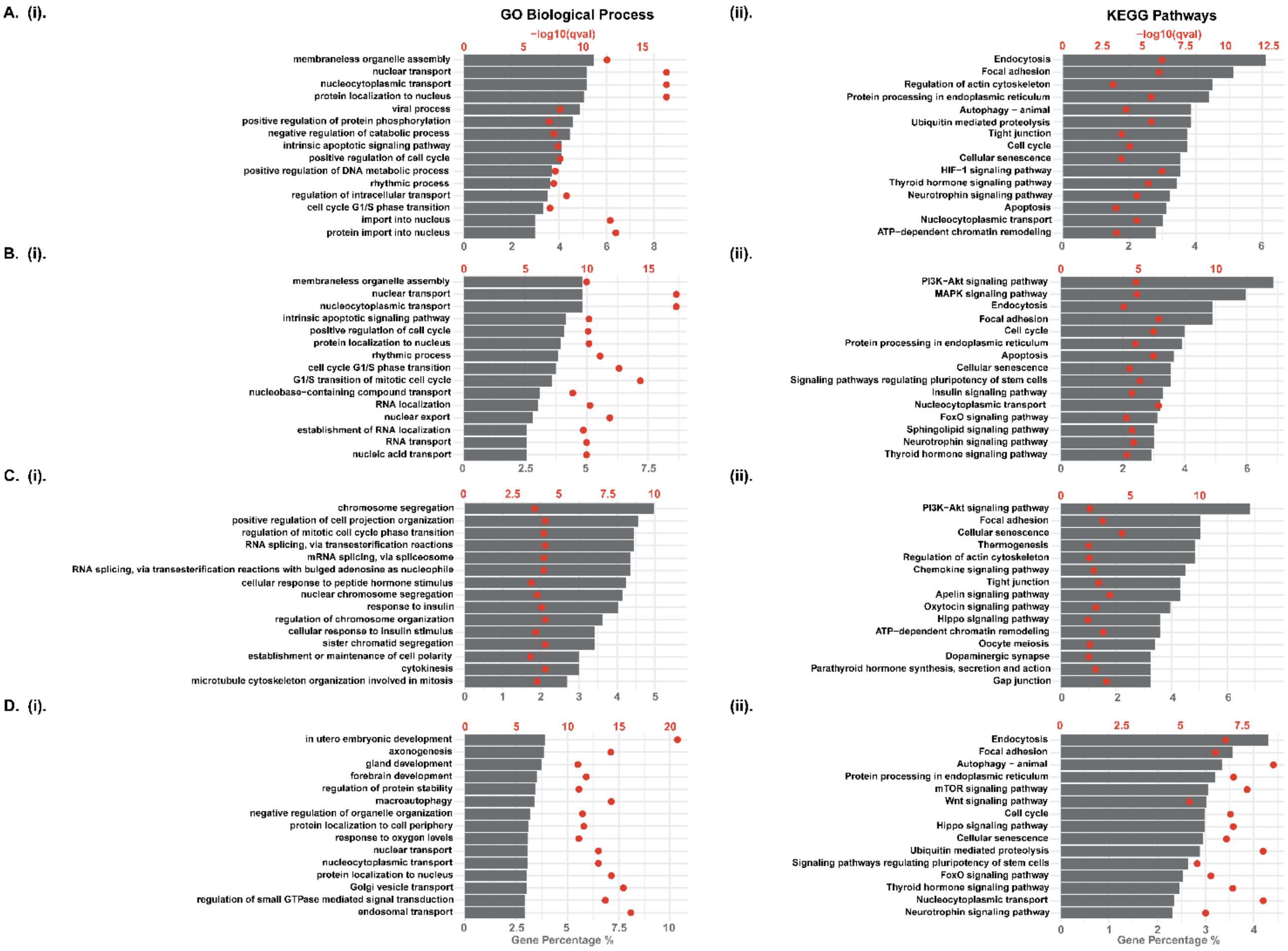

Supplementary Figure S5. Functional enrichment analysis of experimentally validated target genes for selected miRNAs.

Gene Ontology (GO) Biological Process and KEGG pathway enrichment analyses were performed separately for experimentally validated target genes of **(A)** miR-125a-5p, **(B)** miR-125b-5p, **(C)** miR-99b-5p, and **(D)** miR-148a-3p. For each miRNA, panel (i) shows enriched GO Biological Process terms and panel (ii) shows enriched KEGG pathways. Bars represent the percentage (%) of targeted genes involved in each process or function, relative to the total number of genes targeted by the miRNA. Red dots represent the -log10(q-value) of each process or function.
